# The nuclear export protein XPO1 provides a peptide ligand for natural killer cells

**DOI:** 10.1126/sciadv.ado6566

**Published:** 2024-08-23

**Authors:** Matthew D. Blunt, Hayden Fisher, Ralf B. Schittenhelm, Berenice Mbiribindi, Rebecca Fulton, Sajida Khan, Laura Espana-Serrano, Lara V. Graham, Leidy Bastidas-Legarda, Daniel Burns, Sophie M.S. Khakoo, Salah Mansour, Jonathan W. Essex, Rochelle Ayala, Jayajit Das, Anthony W. Purcell, Salim I. Khakoo

**Affiliations:** ^1^ School of Clinical and Experimental Sciences, Faculty of Medicine, University of Southampton, Southampton, UK.; ^2^ School of Cancer Sciences, Faculty of Medicine, University of Southampton, Southampton, UK.; ^3^ School of Chemistry, Faculty of Engineering and Physical Sciences, University of Southampton, Southampton, UK.; ^4^ School of Biological Sciences, Faculty of Environmental and Life Sciences, University of Southampton, Southampton, UK.; ^5^ Monash Proteomics & Metabolomics Platform, Biomedicine Discovery Institute and Department of Biochemistry and Molecular Biology, Monash University, Clayton, Victoria, Australia.; ^6^ Biomedicine Discovery Institute and Department of Biochemistry and Molecular Biology, Monash University, Clayton, Victoria, Australia.; ^7^ Steve and Cindy Rasmussen Institute for Genomic Medicine, Abigail Wexner Research Institute, and The Department of Pediatrics, Pelotonia Institute for Immuno-Oncology, Ohio State University, Columbus, OH, USA.

## Abstract

XPO1 (Exportin-1/CRM1) is a nuclear export protein that is frequently overexpressed in cancer and functions as a driver of oncogenesis. Currently small molecules that target XPO1 are being used in the clinic as anticancer agents. We identify XPO1 as a target for natural killer (NK) cells. Using immunopeptidomics, we have identified a peptide derived from XPO1 that can be recognized by the activating NK cell receptor KIR2DS2 in the context of human leukocyte antigen–C. The peptide can be endogenously processed and presented to activate NK cells specifically through this receptor. Although high XPO1 expression in cancer is commonly associated with a poor prognosis, we show that the outcome of specific cancers, such as hepatocellular carcinoma, can be substantially improved if there is concomitant evidence of NK cell infiltration. We thus identify XPO1 as a bona fide tumor antigen recognized by NK cells that offers an opportunity for a personalized approach to NK cell therapy for solid tumors.

## Teaser

The NK cell receptor KIR2DS2 recognizes a peptide derived from the cancer-associated protein XPO1 in the context of HLA-C.

## INTRODUCTION

Natural killer (NK) cells are becoming increasingly recognized for their anticancer activity, and their functions are tightly controlled by a diverse repertoire of cell surface receptors (*1*). One important family of NK cell receptors are the killer cell immunoglobulin-like receptors (KIRs), which form a polymorphic family of receptors with human leukocyte antigen (HLA) class I ligands (*2*). KIRs have been implicated in susceptibility to, and the outcome of, many different cancers and thus contribute to heterogeneity within the innate immune response to malignancy (*3*–*9*).

The KIR can be activating or inhibitory. The HLA class I ligand specificities of the inhibitory KIR are relatively well defined, while the ligand specificities of the activating KIR have been much harder to identify (*10*). Recent work has shown that activating KIR can have an HLA class I–restricted peptide specificity (*11*–*15*). While T cell receptors have a tight restriction on the peptide:HLA complexes that they bind, KIRs recognize families of peptide:HLA complexes in a motif-based manner allowing recognition of multiple different combination of peptide and HLA (*16*, *17*). We have recently shown that KIR2DS2 recognizes highly conserved flavivirus and hepatitis C peptides with an alanine-threonine sequence at the C-terminal −1 and −2 positions of the peptide in the context of HLA-C (*12*, *18*). We have also recently shown that targeting KIR2DS2 in vivo using a peptide:HLA-C–based strategy can increase NK cell activity against cancer (*19*). In these experiments, KIR2DS2-positive NK cells in the KIR transgenic mouse were activated using a DNA construct that encoded both HLA-C*0102 and the peptide IVDLMCHATF, which together provide a ligand for KIR2DS2.

Activating KIR have been associated with protective responses against a number of cancers, especially hematological malignancies. In genetic studies, KIR haplotypes containing activating KIR confer protection against relapse of hematological malignancies following bone marrow transplantation, and this has been mapped to the region of the *KIR* locus that contains *KIR2DS2* (*20*–*22*). A recent study using sequence-based *KIR* typing has mapped this protection to either the *KIR2DS2* or *KIR2DL2* genes that are in tight linkage disequilibrium within the *KIR* locus (*23*). Furthermore, the benefit of cord blood transplantation is accentuated if the recipient of the transplant has group 1 HLA-C allotypes, the ligands for KIR2DS2 (*24*). *KIR2DS2* has also been associated with protection against a number of solid tumors including cervical neoplasia, breast cancer, lung cancer, colorectal cancer, and hepatocellular carcinoma (HCC) (*4*, *5*, *25*–*27*). These genetic studies lack functional correlates, and although an in vitro study demonstrated recognition of cancer cell lines by KIR2DS2, this was not specific and also encompassed recognition by the inhibitory receptor KIR2DL3 (*28*). As KIR2DS2 recognizes the combination of HLA-C and peptide in a motif-based manner (*12*), this suggests that there is a potential for KIR2DS2 binding peptides to be tumor-associated antigens derived from endogenously expressed proteins that are up-regulated during tumorigenesis. However, to date, no cancer-associated peptides that bind activating KIR have been identified, and we therefore sought to investigate this in a proof-of-concept study.

## RESULTS

### XPO1 provides a peptide recognized by KIR2DS2

**Fig. 1.**
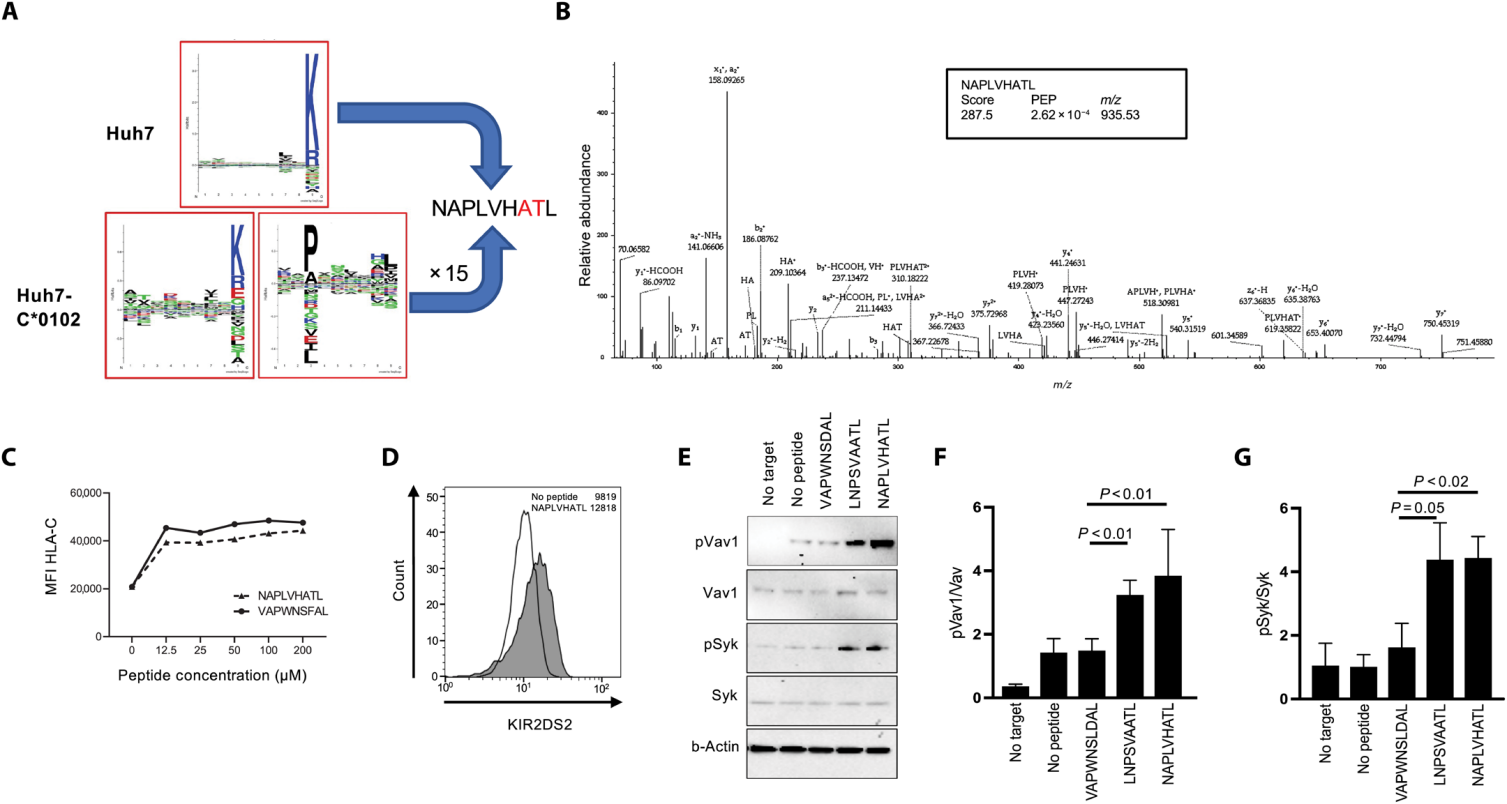
An XPO1-derived peptide is a ligand for KIR2DS2. (**A**) SeqLogo plots of peptides eluted from Huh7 and Huh7:HLA-C*01:02 cell lines. (**B**) Representative and annotated ms2 spectrum that has been assigned to the peptide sequence NAPLVHATL. The resulting Byonic score, the posterior error probability (PEP) and the mass/charge ratio (*m/z*) value of the singly protonated species is shown in the inset. (**C**) 721.174 cells were incubated with NAPLVHATL at the indicated concentrations, stained for HLA-C using the DT9 antibody, and analyzed by flow cytometry. The mean fluorescence intensity (MFI) of DT9 staining compared to the positive control VAPWNSFAL peptide is shown. (**D**) 721.174 cells were incubated with 100 μM NAPLVHATL peptide and stained with a KIR2DS2-tetramer and analyzed by flow cytometry. Histogram plots of KIR2DS2-staining compared with a no peptide control are shown, with median fluorescence intensities indicated. (**E** to **G**) 721.174 cells were incubated overnight with the indicated peptides at 200 μM and cocultured with NKL-2DS2 cells, and then the cells were lysed and analyzed for phosphorylation of Syk (Tyr^323^ and Tyr^317^) and Vav1 (Tyr^174^) by Western blotting. A representative Western blot is shown in (E); the ratios of pVav1 to Vav1 (F) and pSyk to Syk (G) from three independent experiments are also shown. *P* values indicate comparison of the NAPLVHATL peptide with negative (VAPWNSDAL) and positive (LNPSVAATL) control peptides as determined by one-way analysis of variance (ANOVA).

KIR2DS2 is a peptide:HLA-specific receptor, and our previous work had indicated that the human HCC cell line Huh7 transfected with HLA-C*01:02 (Huh7:C1) may be recognized by KIR2DS2 (*19*, *29*). Parental Huh7 cells express HLA-A*11:01, but HLA-B and HLA-C are undetectable on the cell surface (*30*). HLA-A*11:01 binds peptides predominantly with C-terminal lysine or arginine residues and HLA-C*01:02 binds peptides with C-terminal hydrophobic residues. This distinction provided an opportunity for a robust distinction of peptides presented by the two different HLA alleles expressed on the transfected cell line. This allowed identification of potential KIR2DS2-binding peptides expressed on HLA-C*01:02. We sequenced HLA class I–bound peptides from Huh7 and Huh7:C1 cells following elution with the pan class I antibody W6/32. We identified ~5800 and ~7200 peptides from untransfected Huh7 and Huh7:C1 cells, respectively (table S1). As expected, the untransfected Huh7 immunopeptidome consisted mainly of peptides with C-terminal lysine or arginine residues, characteristic of the HLA-A*11:01 binding specificity (Fig. 1A). In contrast, epitopes identified from the Huh7:C1 cell line contained peptides that displayed two distinct sequence motifs, one with the previously observed HLA-A*11:01–binding motif and one with an HLA-C*01:02–binding motif (Fig. 1A). From this comprehensive repertoire of HLA class I binders, only one peptide, NAPLVHATL, was identified that conformed to both the HLA-C*01:02–binding motif (xxPxxxxxL) and the KIR2DS2-binding motif (xxxxxxATx). NAPLVHATL, which was identified by three independent, high-quality peptide-spectrum matches (Fig. 1B), is derived from the nuclear export protein XPO1 (Exportin-1/CRM1). This protein is up-regulated in hematological malignancies and solid tumors including lung cancer, breast cancer, and HCC. It is also associated with a poor prognosis in cancer and is targeted by selinexor, a Food and Drug Administration–approved drug for multiple myeloma and diffuse large B cell lymphoma (*31*–*37*). Data-independent acquisition (DIA) mass spectrometry was subsequently used to quantify NAPLVHATL and other epitopes between the transfected Huh7:C1 and the parental Huh7 cell line (table S2). The peptide NAPLVHATL was eluted at 15-fold greater levels in Huh7:C1 compared to Huh7 cells, consistent with it being presented by endogenous HLA-C*01:02 but at much lower levels. In addition, this peptide has been eluted from HLA class I on cirrhotic livers and a derivative APLVHATL identified from an HCC sample, indicating that this peptide is naturally presented and processed in vivo in the liver (*38*). Furthermore, we did not identify any additional XPO1-derived peptides expressing the HLA-A*1101 motif and, in netMHCpan analysis of the XPO1 protein, there were no predicted HLA-A*1101 peptides with the KIR2DS2-binding motif described by Liu *et al.* (*39*).

To confirm binding to HLA-C*01:02, we cultured transporter associated with antigen processing (TAP)–deficient 721.174 cells, which naturally express HLA-C*01:02, with exogenous NAPLVHATL (*40*). NAPLVHATL stabilized cell surface HLA-C*01:02 molecules to a similar extent to the previously described VAPWNSFAL peptide (*41*) and also bound a KIR2DS2 tetramer (Fig. 1, C and D), confirming that NAPLVHATL is recognized by KIR2DS2 in complex with HLA-C*01:02. To determine whether the NAPLVHATL peptide can activate NK cells, we incubated 721.174 cells overnight with the peptides NAPLVHATL, LNPSVAATL (previously shown to bind KIR2DS2), and VAPWNSDAL (binds HLA-C*01:02 but not KIR2DS2). These were then coincubated with NKL cells transfected with KIR2DS2 (NKL-2DS2), and phosphorylation of Vav1 and Syk was determined. Incubation with the NAPLVHATL peptide led to phosphorylation of both Syk and Vav1, indicating robust activation of the NK cell line (Fig. 1, E to G, and tables S3 and S4).

**Fig. 2.**
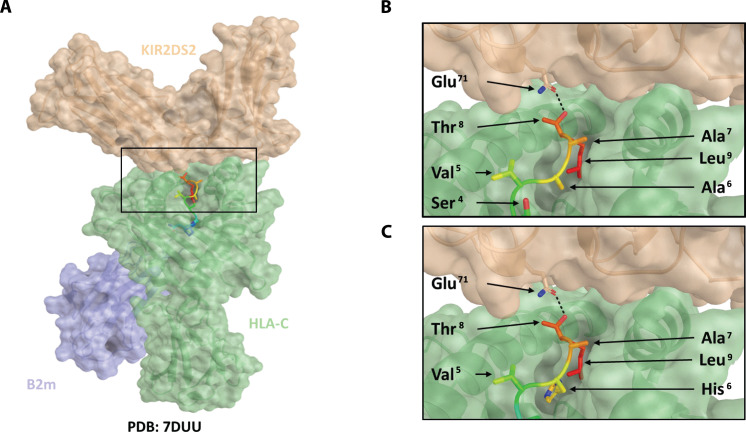
Modeling of HLA-C*01:02, NAPLVHATL, and KIR2DS2 binding interactions. On the basis of the crystal structure of the HLA-KIR complex (PDB: 7DUU), a potential binding mode for the XPO1-derived peptide, NAPLVHATL, was investigated using PyMOL. In silico mutagenesis of the peptide was performed using the protein mutagenesis tool within PyMOL, with the minimization of steric clashes being used to determine the optimal rotameric state of the mutated residue. Peptide-protein interactions were analyzed using PISA. KIR2DS2 is shown as a brown transparent surface, HLA as a green transparent surface, and β2-microglobulin as a blue transparent surface. The peptide is shown as a rainbow ribbon backbone with side chains as sticks. Hydrogen bonds are shown as a black dashed line. (**A**) Model of KIR2DS2 binding with HLA-C*01:02 and NAPLVHATL. Conservation of the KIR2DS2 Glu^71^–peptide Thr^8^ hydrogen bond is shown in (**B**) for the LNPSVAATL crystal structure and in (**C**) for the modeled NAPLVHATL peptide.

The crystal structure of KIR2DS2:HLA-C*01:02 bound with the hepatitis C virus–derived peptide LNPSVAATL has recently been reported (*42*). We therefore used the coordinates and structure factors deposited in the Protein Data Bank (PDB) database (code: 7DUU) to model KIR2DS2 binding with HLAC*01:02 and NAPLVHATL (Fig. 2A). The three C-terminal residues of the peptide are conserved between the structures 7DUU (LNPSVA**ATL**) and NAPLVH**ATL**. Hence, modeling indicated that the binding characteristics of this region are conserved. In the 7DUU crystal structure, an H-bond is formed between Gln^71^ of KIR2DS2 and Thr^8 ^of the peptide (Fig. 2B), and modeling indicated that this key interaction is conserved with the NAPLVHATL peptide, consistent with our observed patterns of NK cell reactivity (Fig. 2C).

### XPO1 recognition by NK cells is KIR2DS2 specific

**Fig. 3.**
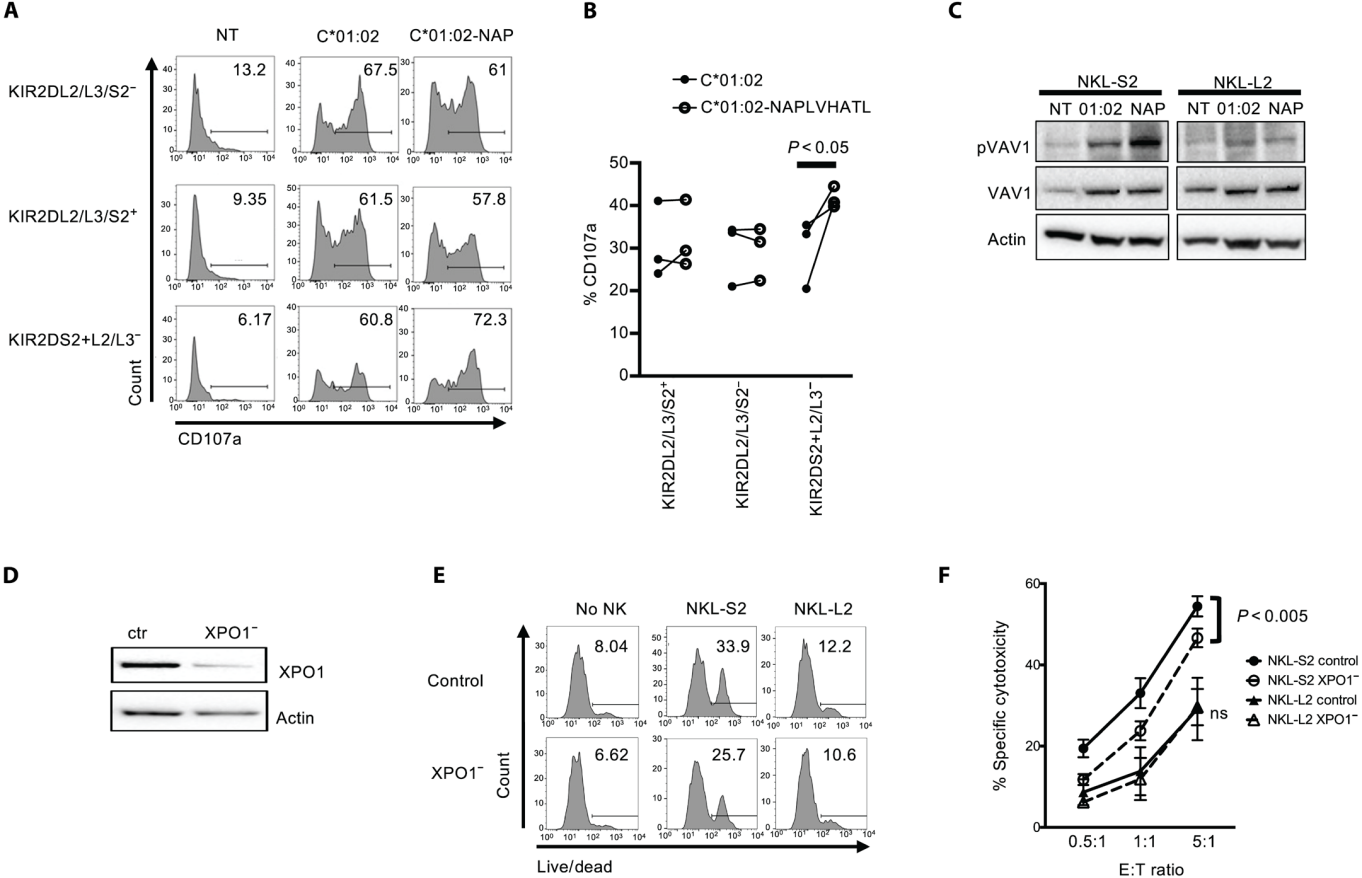
Endogenously presented NAPLVHATL activates KIR2DS2^+^ NK cells. (**A** and **B**) 721.221 cells were transfected with HLA-C*01:02 alone (C*01:02) or in combination with the peptide NAPLVHATL (C*01:02-NAP), and the cell lines were used as targets for degranulation assays with interleukin-15–activated NK cells as effectors. CD107a expression on the indicated subpopulations of CD3^−^CD56^+^ NK cells was assessed by flow cytometry against these targets. (NT = no target). One representative flow cytometry plots of CD107a expression is shown from six healthy donors tested (A), and degranulation from three patients with HCC is shown in (B). *P* values were determined by paired *t* test. (**C**) NKL-2DS2 or NKL-2DL2 cells were incubated with either no target (NT), 721.221:HLA-C*01:02 (C*01:02) or 721.221:HLA-C*01:02+NAPLVHATL (NAP) cells for 5 min and assessed for Vav1 (Tyr^174^) phosphorylation by immunoblotting. A representative image from six experiments is shown. (**D**) Representative immunoblot of XPO1 silencing following XPO1-targeting siRNA treatment of Huh7:HLA-C*01:02 cells. (**E** and **F**) Huh7:HLA-C*01:02 cells were treated with control or XPO1-targeting siRNA and used as targets in cytotoxicity assays with NKL-S2 or NKL-L2 cells. Killing was determined using the LIVE/DEAD stain. Representative plots at an E:T ratio of 5:1 are shown in (E), and the mean specific cytotoxicity and SEM from five independent experiments are shown (F). *P* values were determined by two-way ANOVA.

To determine whether endogenously presented NAPLVHATL was recognized by KIR2DS2-positive NK cells, we created a DNA construct that expressed both HLA-C*01:02 and the peptide NAPLVHATL in a single open reading frame with an intervening T2A self-cleaving sequence (*12*). This was transfected into the HLA class I–negative 721.221 cell line. In CD107a degranulation assays, KIR2DS2^+^/KIR2DL2/3^lo^ NK cells degranulated to a greater extent to the HLA-C*01:02+NAPLVHATL–expressing cell line than to a control cell line expressing only HLA-C*01:02 (Fig. 3A, fig. S1, and table S5), whereas other subpopulations of NK cells, which expressed higher levels of the inhibitory receptors KIR2DL2/3, degranulated at similar levels to both cell lines. This was confirmed in three KIR2DS2-positive patients with HCC (Fig. 3B). In addition, incubation of NKL cells with these 721.221 transfectants led to Vav1 phosphorylation in the NKL-2DS2 cells but not NKL cells transfected with the corresponding inhibitory receptor KIR2DL2 (NKL-2DL2), which interacts with similar HLA-C allotypes to KIR2DS2 (Fig. 3C, fig. S2, and table S6). Thus, NAPLVHATL is recognized by KIR2DS2 in the context of HLA-C*01:02, and this interaction activates NK cells.

To test whether endogenously expressed *XPO1* was recognized by KIR2DS2, we used the Huh7:C1 cell line as a target. XPO1 protein was expressed in these cells and could be knocked down using small interfering RNA (siRNA) (Fig. 3D). In cytotoxicity experiments, NKL-2DS2 cells, but not NKL-2DL2, killed XPO1-knockdown Huh7:C1 cells less efficiently than those transfected with control siRNA at all effector:target (E:T) ratios tested (*P* < 0.001) (Fig. 3, E and F, and table S7). Thus, endogenously expressed *XPO1* is recognized by KIR2DS2^+^ NK cells.

### NK cells enhance survival in XPO1-high cancers

**Fig. 4.**
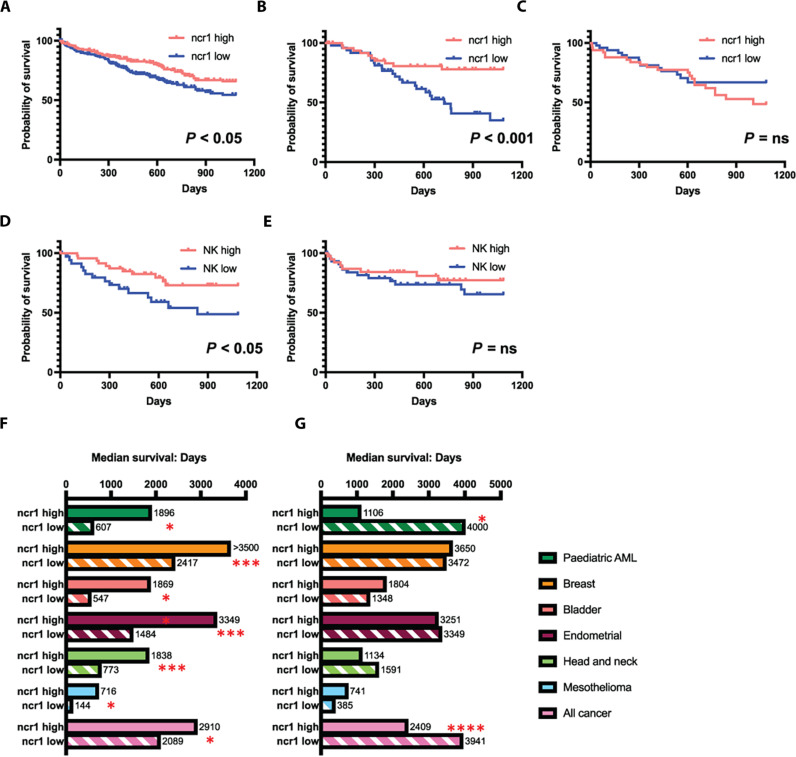
The combination of high levels of XPO1 and *ncr1* expression is associated with improved survival in cancer. (**A** to **C**) Kaplan-Meier plots of HCC TCGA data comparing 3-year survival in individuals with highest (red lines) and lowest (blue lines) quartile levels of *ncr1* expression in the whole cohort (A), individuals with levels of *XPO1* above the median (B), and individuals with levels of *XPO1* below the median (C). (**D** and **E**) show the Kaplan-Meier plots of the *XPO1* high (D) and low (E) analyzed according to peripheral blood NK cell infiltration into the tumors as determined by Cibersort X analysis comparing the highest and lowest quartiles of NK cell infiltration. For all plots, *P* values were calculated using the log-rank Mantel-Cox test. (**F** and **G**) Median survival of individuals with high and low levels of *ncr1* expression in different TCGA datasets comparing individual groups with *XPO1 *levels above (F) and below (G) the median value for each group. *P* values compare the *ncr1^high^* and *ncr1^low^* groups using the log-rank Mantel-Cox test. The following numbers of cases analyzed in each cohort were as follows: pediatric acute myeloid leukemia (AML) (187 cases), breast cancer (1098 cases), bladder cancer (412 cases), endometrial cancer (559 cases), head and neck cancer (528 cases), mesothelioma (86 cases), and all cancers (11,768 cases). *P < 0.05, **P < 0.01, ***P < 0.005, ****P < 0.0001.

To identify whether *XPO1* expression recognition by NK cells has clinical relevance for HCC, we queried the Genomic Data Commons (GDC) Cancer Genome Atlas (TCGA) database using the XENA tool (*43*). This contains 377 cases of HCC with RNA expression data (*44*). As *KIR2DS2 *is not identified using conventional RNA sequencing (RNA-seq) methodologies because of a paucity of unique reads, we used the NK cell lineage defining marker *ncr1*, which encodes NKp46, to identify tumors with higher infiltration of NK cells. In the overall cohort, median survival was similar between the *ncr1^high^* and *ncr1^low^* groups at 1624 and 1622 days, respectively. However, 3-year survival was significantly longer in the *ncr1^high^* group, *P* = 0.024 (Fig. 4A). We therefore analyzed the data for the combination of XPO1 and *ncr1* expression. In individuals with levels of *XPO1* expression above the median, NK cell infiltration was associated with improved long-term survival of individuals with HCC at 3 years (Fig. 4B and Table 1). This was also present at 2 (*P* < 0.018), 4 (*P* < 0.015), and 5 years (*P* < 0.029). Median survival in individuals with higher *XPO1* levels was 2131 days if they also had high levels of *ncr1* expression, compared to 765 in individuals with low levels of *ncr1* expression. However, there was no survival benefit of NK cell infiltration in individuals with lower levels of *XPO1* expression at any time point, and median survival was more similar, in both groups 1005 (*ncr1^high^*) versus 1964 (*ncr1^low^*) (Fig. 4C). Survival benefit in HCC was also associated with a number of other key NK cell–associated genes including *NKG7*, *KLRK1* (NKG2D), *KLRB1* (CD94), *KLRD1* (CD161), and *KLRF1* (NKp80), thus excluding innate lymphoid cells as a potential confounding variable for *ncr1* expression (Table 1) (*45*). Cibersort X analysis permits deconvolution of bulk RNA-seq data from complex mixtures of cells into distinct lymphocyte subpopulations (*46*). We used RNA-seq data from studies of peripheral and intrahepatic NK cells to derive a specific NK cell matrix consisting of peripheral NK cells from healthy donors, peripheral CD49a-activated NK cells, and intrahepatic CXCR6-positive and intrahepatic CXCR6-negative cells (*47*, *48*). This matrix was then used to impute the relative fractions of NK cells from the GDC TCGA HCC RNA-seq dataset (table S8). In the *XPO1^high^* group, the presence of the subpopulation of healthy peripheral NK cells within HCCs was associated with a trend toward a longer median survival in the *XPO1^high^* group (median survival 1490 days versus 837 days, *P* = 0.15). Peripheral NK cells were also associated with better 3-year survival in tumors with higher levels of *XPO1* (*P* < 0.05 log-rank test) but not in those with lower levels of *XPO1* (*P* > 0.1) (Fig. 4, D and E). No correlations were found with survival and the other subpopulations of NK cells interrogated. Thus, intratumoral NK cells are associated with a beneficial outcome of HCC in individuals with higher levels of *XPO1*, indicating an opportunity for stratification of patients with HCC for NK cell therapy.

**Table 1.** *P* values of Kaplan-Meier survival analyses of the GDC TCGA HCC dataset (377 samples) performed for the indicated NK cell genes. HCC samples for the GDC TCGA cohort were categorized into high and low *XPO1* levels on the basis of median expression. Shown are the *P* values for the Kaplan-Meier curves between groups expressing high and low levels of the indicated NK cell genes, in each of the XPO1 categories, indicating an association of expression of NK cell genes with long-term survival.

NK cell gene	XPO1 level	1-year survival	2-year survival	3-year survival	4-year survival	5-year survival
***Ncr1* (NKp46)**	High	0.296	0.018	0.005	0.015	0.029
	Low	0.731	0.672	0.915	0.599	0.478
***NKG7***	High	0.006	0.003	0.0007	0.001	0.002
	Low	0.252	0.983	0.694	0.377	0.889
***KLRB1 *(CD161)**	High	0.008	0.003	0.002	0.009	0.0089
	Low	0.589	0.942	0.217	0.341	0.505
***KLRD1* (CD94)**	High	0.068	0.026	0.005	0.015	0.032
	Low	0.646	0.986	0.544	0.368	0.223
***KLRF1 *(NKp80)**	High	0.007	0.002	0.002	0.001	0.002
	Low	0.720	0.831	0.562	0.300	0.109
***KLRK1 *(NKG2D)**	High	0.030	0.006	0.002	0.003	0.005
	Low	0.814	0.669	0.560	0.366	0.328

As *XPO1* is overexpressed in many different cancers, we used the same methodology to query all the cancer data in the GDC database (*49*). Data was available for 11,768 individuals in the pan-Cancer cohort, and these showed that high levels of *ncr1* expression were associated with an enhanced median survival of 2910 days versus 2089 days, *P* = 0.017 in individuals with higher levels of *XPO1*. However, in individuals with lower levels of *XPO1*, *ncr1* expression was associated with worse median survival 2049 versus 3941 days, *P* < 0.0001 (Fig. 4, F and G). To identify which other cancers had a positive association between *XPO1*, *ncr1*, and survival, we interrogated the 39 different GDC TCGA datasets covering all the cancer types in the TCGA database. Of these, there were positive associations with bladder cancer (412 cases, median survival *ncr1^high^* 1869 versus 570 days *ncr1^low^*, *P* = 0.0087); breast cancer (1098 cases median survival *ncr1^high^* > 3650 versus 2712 days *ncr1^low^*, *P* = 0.0253); endometrial cancer (559 cases, median survival *ncr1^high^* 3349 versus 1484 days *ncr1^low^*, *P* = 0.0041); head and neck cancer (528 cases, median survival *ncr1^high^* 1838 versus 773 days *ncr1^low^*, *P* = 0.0024); mesothelioma (86 cases, median survival *ncr1^high^* 715.5 versus 144 days *ncr1^low^*, *P* = 0.047); and pediatric acute myeloid leukemia (AML) (187 cases, median survival *ncr1^high^* 1896 versus 607 days *ncr1^low^*, *P* = 0.016). High *ncr1* expression was not positively associated with survival in the XPO1-low groups, and in the pediatric AML cohort, it was associated with worse survival (1106 days versus > 3650 days, *P* = 0.053). Thus, the combination of high levels of both XPO1 and NK cell infiltration exerts a cancer-specific protective effect on long-term survival in cancer.

## DISCUSSION

Substantial evidence demonstrates the association of *KIR2DS2* with protection against cancer (*9*, *24*). We now describe a molecular mechanism underlying a KIR2DS2 specific response against cancer and identify that, in multiple cancers, the combination of XPO1 and NK cells are associated with longer survival. However, within the TCGA dataset, we were unable to determine whether it was specifically *KIR2DS2* that associated with *XPO1* in prolonging survival, and this will require further detailed investigation. Previous work has shown that the KIR family of NK cell receptors provides diversity in the innate immune response to cancer. Immunogenetic studies have identified that *KIR2DS2* may be associated with beneficial responses to a number of different malignancies. The identification of a naturally processed and presented KIR2DS2-binding peptide provides a potential mechanism for this genetic association. The peptide we identified, NAPLVHATL, is derived from XPO1 and was the only peptide that we identified from the peptidomes we sequenced that contained the canonical KIR2DS2-binding AT motif at the C-terminal −1 and −2 positions. XPO1 is thought to function as the sole nuclear exporter for many different tumor suppressor and growth-regulatory proteins including p53, p27, FOXO1, IkB, cyclin B1, cyclin D1, and survivin (*31*). It is overexpressed and associated with poor prognosis in multiple cancers including hematological malignancies and difficult to treat cancers such as pancreatic cancer and HCC (*34*, *36*, *50*). Furthermore, following clinical trials, inhibition of XPO1 is gaining traction as a cancer therapeutic target and is now licensed for multiple myeloma and diffuse large B cell lymphoma (*37*). Thus, this protein has an important role in both the pathogenesis and treatment of cancer.

In healthy cells, XPO1 is expressed at low levels, which we speculate are too low to generate an activating signal in KIR2DS2-positive NK cells. The NK cell activation threshold is complex, combining education of NK cells by interactions between inhibitory receptors and HLA class I, and different combinations of signals from different activating stimuli (*51*, *52*). These prevent NK cells from being autoreactive while maintaining reactivity against stressed or diseased cells. We propose that the up-regulation of XPO1 found in cancer can alter the balance in favor of activation of KIR2DS2-positive NK cells. Consistent with this, knocking down XPO1 led to a decrease in killing of Huh7:C1 targets. However, to comprehensively prove that the NAPLVHATL epitope is being endogenously presented and is solely responsible for recognition by KIR2DS2-positive NK cells would require further experiments using CRISPR editing of the NAPLVHATL epitope within the Huh7:C1 cells.

Previous work has shown that KIR2DS2^+^ NK cells can recognize several different cancer targets in vitro, including cell lines derived from prostate, breast, and ovarian carcinomas (*28*). However, this recognition was not specific to KIR2DS2 and was β2-microglobulin independent. This suggests that there may also be non-peptide:HLA class I ligands for KIR2DS2, in addition to the cancer-associated NAPLVHATL peptide derived from XPO1. Furthermore, in this study, the same cell lines were also recognized by both KIR2DS2 and KIR2DL2, indicating that this unidentified ligand is not specific for KIR2DS2.

KIR2DS2 and its inhibitory counterparts KIR2DL2 and KIR2DL3 all bind HLA-C*0102. In our experiments, we did not identify any increased inhibition by NAPLVHATL mediated through KIR2DL2/3 in experiments using human peripheral blood mononuclear cells (PBMCs) or the NKL-2DL2 transfectant. This may be because these cells are already maximally inhibited by HLA-C*0102. In structural studies, KIR2DL2 preferentially binds peptides with large hydrophobic amino acids at P7, which interact directly with residues 104 and 105 of KIR2DS2, and small amino acids at P8 (*53*), and alanine. Therefore, although we cannot exclude binding of KIR2DL2 and KIR2DL3 to HLA-C*0102 in complex with NAPLVHATL, it is unlikely to be a strong binder. Consistent with this, there was no change in inhibition of NKL-2DL2 cells when XPO1 was knocked down.

KIR2DS2^+^ NK cells express a transcriptional profile associated with augmented NK cell–mediated cytotoxicity and display a greater potential for antibody-dependent cellular cytotoxicity (ADCC) in vitro and in vivo (*54*–*56*). Consistent with the generalized enhanced activity of KIR2DS2-positive NK cells, KIR2DS2 and its cognate group 1 HLA-C ligands have been associated with beneficial outcomes in viral infections such as HCV and severe acute respiratory syndrome coronavirus 2 and is associated with autoimmune disorders (*10*, *12*, *57*). Thus, there are a number of different rationales for targeting the KIR2DS2^+^ subset of NK cells for immunotherapy. NK cell immunotherapy is currently an exciting area, with adoptive transfer of NK cells activated in vitro and CAR-NK cells and NK cell engagers at the forefront of clinical trials. Our data show that KIR2DS2^+^ NK cells represent a target for cancer immunotherapy, which could be addressed either through a CAR-NK cell approach or through direct activation of KIR2DS2-positive NK cells. The high sequence homology between KIR2DS2 and its inhibitory counterparts KIR2DL2 and KIR2DL3 has meant that raising antibodies to specifically target KIR2DS2 is especially challenging. However, we have recently shown that a construct encoding the peptide:HLA ligand for KIR2DS2 can specifically augment the anticancer activity of KIR2DS2-positive NK cells and so presents an alternative immunotherapeutic strategy (*19*). Thus, we propose a peptide:HLA-based mechanism for how NK cells may recognize malignant cells and highlight the potential for KIR2DS2 as a target for cancer immunotherapy.

## MATERIALS AND METHODS

### Peptide elution, identification, and quantification

HLA class I peptides were eluted from Huh7 and Huh7:HLA-C*01:02 cells as previously described (*58*–*60*). In brief, HLA-peptide eluates were loaded onto a C18 RP-HPLC column (Chromolith Speed Rod; Merck), and the bound peptides were separated using increasing concentration of 80% acetonitrile, 0.1% trifluoroacetic acid (80% ACN/0.1% TFA). Peptide-containing fractions were collected and reconstituted with 0.1% formic acid (FA). Indexed retention time peptides were spiked in for retention time alignment. For peptide identification, samples were then loaded via an Acclaim PepMap 100 trap column (Thermo Fisher Scientific) onto an Acclaim PepMap RSLC analytical column (Thermo Fisher Scientific) using a Dionex UltiMate 3000 RSLCnano system (Thermo Fisher Scientific), separated using increasing concentrations of 80% ACN/0.1% FA and analyzed with a Q Exactive mass spectrometer (Thermo Fisher Scientific). For quantification, HLA-bound peptides were analyzed on an Orbitrap Fusion Tribrid mass spectrometer (Thermo Fisher Scientific) coupled to an identical liquid chromatography setup using DIA. Fifty sequential DIA windows with an isolation width of 12 mass/charge ratio (*m/z*) between 375 and 975 *m*/*z* were acquired in 2 consecutive injections following a full ms1 scan (resolution, 120.000; Automatic Gain Control (AGC) target, 4 × 10^5^; maximum ion trap (IT), 50 ms; scan range, 375 to 1575 *m*/*z*). Acquired DDA .raw files were searched against the human UniProtKB/SwissProt database (v2012_07) using Byonic (Protein Metrics) embedded in Proteome Discoverer (Thermo Fisher Scientific) to obtain peptide sequence information. Only peptides identified at a false discovery rate of 1% based on a decoy database were considered for further analysis. Spectronaut Orion (Biognosys) was used to create the corresponding spectral library as well as to evaluate all DIA data using in-house, peptide-centric parameters.

### Cell lines

HLA class I–deficient 721.221 lymphoblastoid Epstein-Barr virus–B cells were cultured in R10 medium [RPMI 1640 supplemented with 1% penicillin-streptomycin (Life Technologies) and 10% heat-inactivated fetal bovine serum (Sigma-Aldrich)]. 721.221 cells were transduced with the pIB2 constructs to express HLA-C*01:02 alone or together with peptide NAPLVHATL. For tetramer staining, 721.174 cells were cultured in R10 incubated overnight with peptides and then stained with KIR2DS2 tetramers as previously described (*12*).

### Flow cytometry analyses

Human PBMCs were obtained, with informed consent and full ethical approval from the National Research Ethics Committee references 06/Q1701/120 (patients with HCC) and 19/WM/0262 (healthy volunteer samples). PBMCs stimulated with interleukin-15 (1 ng/ml) overnight were incubated with 721.221 or Huh7:HLA-C*01:02 cells for 4 hours, anti-CD107a-AF647 (Biolegend) with GolgiStop added 1 hour after coculture, and cells analyzed by flow cytometry. For Huh7:HLA-C*01:02 cytotoxicity assays, target cells were coincubated with the indicated NK cell population for 4 hours. Cells were then stained with LIVE/DEAD stain (Thermo Fisher Scientific) and analyzed by flow cytometry, gating on the target cell population identifiable by green fluorescent protein within the HLA-C*01:02 construct. For XPO1 knockdown, Huh7:HLA-C*01:02 cells were transfected using Jetprime (Polyplus, UK) reagent with siRNA control or siRNA targeting XPO1 (Thermo Fisher Scientific). XPO1 expression was analyzed by immunoblotting after 48 hours, and cells were used in cytotoxicity and degranulation assays 48 hours after transfection.

### Immunoblotting

NKL-KIR2DS2 or NKL-KIR2DL2 was coincubated with 721.174 cells incubated with 200 mM peptide, 721.221:HLA-C*01:02, or 721.221:HLA-C*01:02+NAPLVHATL cells, as indicated for 5 min at an E:T ratio of 1:1. Cells were then lysed in NP40 Cell Lysis Buffer (Thermo Fisher Scientific UK Ltd) and analyzed by immunoblotting. Antibodies recognizing phospho-Syk, Syk, phospho-Vav1, Vav1, and actin (Thermo Fisher Scientific) were used with horseradish peroxidase–conjugated antibodies and visualized using the ChemiDoc-It Imaging system (Bio-Rad). Bands were quantified using ImageJ software.

### Structure analysis and in silico mutagenesis

Analysis of the crystal structure of the KIR2DS2-HLA-C-peptide complex (PDB: 7DUU) was performed in PyMOL (Version 2.5 Schrödinger, LLC). In silico mutagenesis of the peptide was performed using the protein mutagenesis tool within PyMOL, with the minimization of steric clashes being used to determine the optimal rotameric state of the mutated residue. Peptide-protein interactions were analyzed using PISA (*61*).

### TCGA analysis

Datasets from the GDC TCGA data hub were analyzed. HCC data were downloaded from https://gdc-hub.s3.us-east-1.amazonaws.com/download/TCGA-LIHC.htseq_counts.tsv.gz, and survival analysis performed using Graph-Pad Prism 7.0 software. The following datasets were interrogated: GDC Pan-Cancer, GDC TARGET-ALL-P3, GDC TARGET-AML, GDC TARGET-CCSK, GDC TARGET-NBL, GDC TARGET-OSGDC, TARGET-RT, GDC TARGET-WT, GDC TCGA Acute Myeloid Leukemia, GDC TCGA Adrenocortical Cancer, GDC TCGA Bile Duct Cancer, GDC TCGA Bladder Cancer, GDC TCGA Breast Cancer, GDC TCGA Cervical Cancer, GDC TCGA Colon Cancer, GDC TCGA Endometrioid Cancer, GDC TCGA Esophageal Cancer, GDC TCGA Glioblastoma, GDC TCGA Head and Neck Cancer, GDC TCGA Kidney Chromophobe, GDC TCGA Kidney Clear Cell Carcinoma, GDC TCGA Kidney Papillary Cell Carcinoma, GDC TCGA Large B-cell Lymphoma, GDC TCGA Liver Cancer (HCC cases only), GDC TCGA Lower Grade Glioma, GDC TCGA Lung Adenocarcinoma, GDC TCGA Lung Squamous Cell Carcinoma, GDC TCGA Melanoma, GDC TCGA Mesothelioma, GDC TCGA Ocular melanomas, GDC TCGA Ovarian Cancer, GDC TCGA Pancreatic Cancer, GDC TCGA Pheochromocytoma & Paraganglioma, GDC TCGA Prostate Cancer, GDC TCGA Rectal Cancer, GDC TCGA Sarcoma, GDC TCGA Stomach Cancer, GDC TCGA Testicular Cancer, GDC TCGA Thymoma, GDC TCGA Thyroid Cancer, and GDC TCGA Uterine Carcinosarcoma.

Cibersort X analysis was performed using an NK cell matrix derived from RNA-seq data of intrahepatic (CXCR6^+^ and CXCR6^−^) and peripheral (CD49a^+^ and CD49a^−^) NK cell subpopulations, which was used to query GDC TCGA Liver Cancer HCC dataset (*46*–*48*). For all survival analysis, the highest and lowest quartiles of NK cell expression were compared.

### Statistical analysis

Experimental statistical analyses were performed using GraphPad Prism 7.0 software. Student’s two-tailed *t* test was used for comparison between two groups, and two-way analysis of variance (ANOVA) with post hoc analysis were used to compare more than two groups. Data were considered statistically significant at *P* < 0.05. Survival levels were determined using the log-rank Mantel-Cox method.
