# Supplementary material for: The nuclear export protein XPO1 provides a peptide ligand for natural killer cells
Source: Sci Adv. 2024 Aug 23;10(34):eado6566. doi: 10.1126/sciadv.ado6566 (PMC11343027; doi:10.1126/sciadv.ado6566)
Supplement: Supplementary file 1 — Figs. S1 and S2 Tables S3 to S7 Legends for tables S1, S2, and S8 [file sciadv.ado6566_sm.pdf]

Science Advances
AAAS

Supplementary Materials for

The nuclear export protein XPO1 provides a peptide ligand for natural killer cells

Matthew D. Blunt *et al.*

Corresponding author: Salim I. Khakoo, s.i.khakoo@soton.ac.uk

Sci. Adv. **10**, eado6566 (2024)
DOI: 10.1126/sciadv.ado6566

The PDF file includes:

Figs. S1 and S2
Tables S3 to S7
Legends for tables S1, S2, and S8

Other Supplementary Material for this manuscript includes the following:

Tables S1, S2, and S8

Supplementary figure S1: KIR2DS2^{high} NK cells are activated by cells expressing HLA-C*0102 in combination with the NAPLVHATL peptide

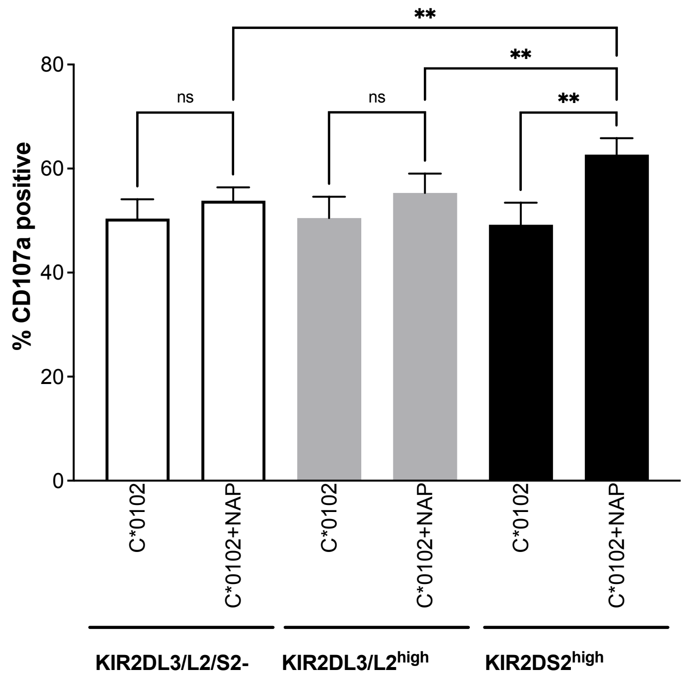

721.221 cells were transfected with HLA-C*01:02 alone (C*0102) or in combination with the peptide NAPLVHATL (C*0102+NAP), and the cell lines used as targets for degranulation assays with IL-15 activated NK cells as effectors. CD107a expression on the indicated sub-populations of CD3-CD56+ NK cells was assessed by flow cytometry against these targets. Summary results from the six donors tested are shown (**p<0.01; ANOVA with Tukey's test for multiple comparisons).

Figure S2: VAV1 phosphorylation of 2DS2-positive NKL cells by NAPLVHATL

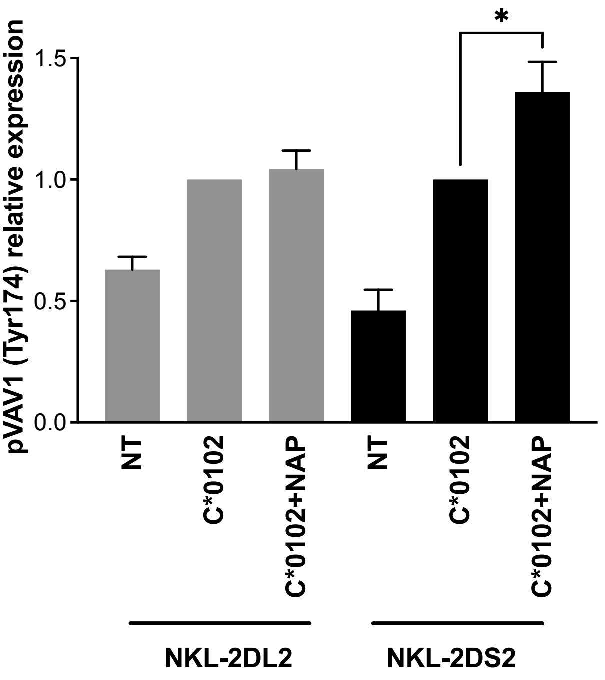

NKL-2DS2 or NKL-2DL2 cells were incubated with either no target (NT), 721.221:HLA-C*01:02 (C*0102) or 721.221:HLA-C*0102+NAPLVHATL (C*0102+NAP) cells for 5 minutes and assessed for Vav1 (Tyr174) phosphorylation by immunoblotting, quantified using the ChemiDoc-It Imaging system. Means and standard deviations and standard errors are shown. The NAPLVHATL induces activation of NKL-2DS2 cells but does not change the level of activation of NKL-2DL2 cells (*$p<0.05$: paired t-test).

Supplementary Tables:

Supplementary Table S1: Peptides identified by mass spectrometry from Huh7 and Huh7:C1 cells (accompanying xls file)

Supplementary Table S2: Quantitation of peptides eluted from Huh7 and Huh7:C1 cells (accompanying xls file)

Supplementary Table S3: Western blot densitometry ratios of pVav1/Vav1 in NKL-2DS2 cells following incubation with the different peptides in Figure 1F

Experiment	No target	No peptide	VAPWNSDAL	LNPSVAATL	NAPLVHATL
1	0.437	0.86158	1.274471	3.04495	5.520958
2	0.239377	1.74814	1.922124	2.844377	2.801711
3	0.325415	1.582443	1.1894527	3.762356	3.12053

Supplementary Table S4: Western blot densitometry ratios of pSyk/Syk in NKL-2DS2 cells following incubation with the different peptides in Figure 1G

Experiment	No target	No peptide	VAPWNSDAL	LNPSVAATL	NAPLVHATL
1	1.808348	1.440003	2.231815	5.684421	5.099912
2	0.366667	0.655251	0.723082	3.968966	3.689665
3	0.8953106	0.8542156	1.8346883	3.426789	4.42731

Supplementary Table S5: CD107a degranulation on CD3-CD56+ NK cells for Figure 3A and Figure S1. Donors were tested against 721.221 cells expressing HLA-C*0102 (C*0102) or HLA-C*0102 plus NAPLVHATL (C*0102+ NAPLVHATL)

**Sub-population:**	**KIR2DL3/L2/S2-negative**		**KIR2DL3/2hi**		**KIR2DS2hi**	
**Target:**	C*0102	C*0102+ NAPLVHATL	C*0102	C*0102+ NAPLVHATL	C*0102	C*0102+ NAPLVHATL
**Donor 1**	31.03	44.23	26.46	47.26	36.03	61.73
**Donor 1**	31.83	46.23	26.66	46.36	37.03	62.33
**Donor 2**	40.89	44.39	44.18	36.88	27.46	44.76
**Donor 2**	39.29	43.69	45.98	35.58	30.06	44.96
**Donor 3**	55.4	56.2	52.15	57.45	62.73	64.73
**Donor 3**	54.3	55.6	52.85	57.55	54.63	66.03
**Donor 4**	57.49	57.69	59.51	60.01	55.04	60.74
**Donor 5**	58.11	63.21	58.97	69.17	52.63	70.63
**Donor 5**	56.51	57.91	59.27	61.87	55.23	65.73
**Donor 6**	65.1	69.6	64.6	72.9	62.65	82.05
**Donor 6**	64.2	53.4	64.9	63.6	67.85	65.75

Supplementary Table S6: Western blot densitometry data of pVav1 (Tyr174) levels in NKL-2DL2 or NKL-2DS2 cells incubated with 721.221 cells expressing HLA-C*0102 (C*0102) or expressing HLA-C*0102 plus NAPLVHATL as shown in Figure 3C and Figure S2. Data are normalised to C*0102 cells.

**NKL-2DL2**				**NKL-2DS2**			
**Experiment**	**No Target**	**C*0102**	**C*0102+ NAPLVHATL**	**Experiment**	**No Target**	**C*0102**	**C*0102+ NAPLVHATL**
1	0.605537	1	**0.935796**	2	0.47808	1	1.306768
3	0.738612	1	**0.948743**	4	0.424295	1	1.073959
5	0.678817	1	**1.02544**	6	0.254012	1	1.814331
7	0.495923	1	**1.263223361**	8	0.235058	1	1.132993
				9	0.789061	1	1.649083
				10	0.585369	1	1.190711

Supplementary Table S7: Cytotoxicity data for Figure 3F showing the specific cytotoxicity of NKL-2DS2 and NKL-2DL2 against Huh7:C1 cells transfected with siRNA to XPO1 or control siRNA

	NKL-2DS2										NKL-2DL2								
**E:T ratio**	**Control siRNA**					**XPO1 siRNA**					**Control siRNA**					**XPO1 siRNA**			
**0.5:1**	22.9	23.3		14.59	16.96	14.67	13.38		9.78	9.28	13.4	14.8		2.19	4.26	8.57	8.08		4.08
**1:1**	36.4	34.5	44.29	24.19	25.86	25.07	24.88	31.31	18.58	19.08	12.4	13	36.09	3.49	4.16	10.67	8.68	32.01	4.28
**5:1**	58.7	59.2	45.39	53.59	55.26	52.67	51.08	40.31	44.38	44.78	42.1	41.7	41.29	10.29	10.36	37.97	35.58	37.11	18.58

Supplementary Table S8: Cibersort X analysis of hepatocellular carcinoma samples from TCGA LIHC dataset (accompanying xls file)
